# Testing the Community-Based Learning Collaborative (CBLC) implementation model: a study protocol

**DOI:** 10.1186/s13033-016-0084-4

**Published:** 2016-08-18

**Authors:** Rochelle F. Hanson, Sonja Schoenwald, Benjamin E. Saunders, Jason Chapman, Lawrence A. Palinkas, Angela D. Moreland, Alex Dopp

**Affiliations:** 1 Department of Psychiatry and Behavioral Sciences, National Crime Victims Research and Treatment Center, Medical University of South Carolina, 67 President Street, MSC 861, Charleston, SC USA; 2 Division of Global and Community Health, Department of Psychiatry and Behavioral Sciences, Medical University of South Carolina, Charleston, SC USA; 3 Oregon Social Learning Center, 10 Shelton McMurphey Blvd, Eugene, OR 97401 USA; 4 School of Social Work, University of Southern California, 669W. 34th Street, MC0411, Los Angeles, CA 90089-0411 USA

**Keywords:** Implementation, Evidence-based treatment, Youth violence exposure, Learning collaboratives, Interprofessional collaboration

## Abstract

**Background:**

High rates of youth exposure to violence, either through direct victimization or witnessing, result in significant health/mental health consequences and high associated lifetime costs. Evidence-based treatments (EBTs), such as *Trauma*-*Focused Cognitive Behavioral Therapy* (TF-CBT), can prevent and/or reduce these negative effects, yet these treatments are not standard practice for therapists working with children identified by child welfare or mental health systems as needing services. While research indicates that collaboration among child welfare and mental health services sectors improves availability and sustainment of EBTs for children, few implementation strategies designed specifically to promote and sustain inter-professional collaboration (IC) and inter-organizational relationships (IOR) have undergone empirical investigation. A potential candidate for evaluation is the Community-Based Learning Collaborative (CBLC) implementation model, an adaptation of the Learning Collaborative which includes strategies designed to develop and strengthen inter-professional relationships between brokers and providers of mental health services to promote IC and IOR and achieve sustained implementation of EBTs for children within a community.

**Methods/design:**

This non-experimental, mixed methods study involves two phases: (1) analysis of existing prospective quantitative and qualitative quality improvement and project evaluation data collected pre and post, weekly, and monthly from 998 participants in one of seven CBLCs conducted as part of a statewide initiative; and (2) Phase 2 collection of new quantitative and qualitative (key informant interviews) data during the funded study period to evaluate changes in relations among IC, IOR, social networks and the penetration and sustainment of TF-CBT in targeted communities. Recruitment for Phase 2 is from the pool of 998 CBLC participants to achieve a targeted enrollment of *n* = 150. Study aims include: (1) Use existing quality improvement (weekly/monthly online surveys; pre-post surveys; interviews) and newly collected quantitative (monthly surveys) and qualitative (key informant interviews) data and social network analysis to test whether CBLC strategies are associated with penetration and sustainment of TF-CBT; and (2) Use existing quantitative quality improvement (weekly/monthly on-line surveys; pre/post surveys) and newly collected qualitative (key informant interviews) data and social network analysis to test whether CBLC strategies are associated with increased IOR and IC intensity.

**Discussion:**

The proposed research leverages an on-going, statewide implementation initiative to generate evidence about implementation strategies needed to make trauma-focused EBTs more accessible to children. This study also provides feasibility data to inform an effectiveness trial that will utilize a time-series design to rigorously evaluate the CBLC model as a mechanism to improve access and sustained use of EBTs for children.

## Background

High rates of youth exposure to violence, either through direct victimization or witnessing, represent a global public health crisis [1–7]. In the United States, an estimated 40–80 % of children and adolescents experience some type of traumatic event in their lifetime [8]. Rates of exposure to potentially traumatic events are particularly high among children in foster care, with estimates at 90 % [9]. Given that children exposed to potentially traumatic incidents are at risk for myriad short and long term physical and mental health problems, it is essential to maximize access to trauma-focused evidence-based treatments (EBTs) [10–15].

Rigorous research has identified a number of trauma-focused EBTs, with Trauma-focused Cognitive Behavioral Therapy (TF-CBT) [16] having the most empirical support [17–22]. With the aim of efficiently advancing the larger scale implementation in routine care of TF-CBT (and other trauma focused EBTs), the National Child Traumatic Stress Network, funded by the Substance Abuse Mental Health Services Administration since 2000, has promulgated use of the Learning Collaborative (LC) implementation model [23, 24]. The LC model [25, 26] brings together teams from different organizations to work together to learn an EBT and sustain its use over time. In a review of pertinent research, Nadeem et al. [27] identified a number of ‘cross-cutting’ LC elements, including in-person training sessions, telephone consultation groups, data reporting, leadership involvement, and training in quality improvement methods (e.g., Plan-do-Study-Act cycles, multidisciplinary quality improvement teams).

Research on the nature, efficiency, and effectiveness of LC approaches to implementation, however, is limited. For instance, core LC components were often poorly defined and measured in previous studies, making it difficult to determine which ingredients promoted positive provider and patient outcomes [27]. Accordingly, it was recommended that future studies include clear definitions of LC components and the means by which to measure those components [28–31]. In addition, although Nadeem et al’s review [27] concluded that LCs hold “promise for increasing sustained change by building local capacity and for addressing organization and provider-level variables…” (p 383); and have the potential to create an ‘*inter*-*organizational support network*’ to share and learn from others’ successes and challenges (p 384), studies have not yet examined the degree to which these models influence interprofessional relationships across multiple service sectors [31–35], nor whether these relationships result in the increased use of EBTs for children. Indeed, empirical evaluation of implementation models, such as the LC, is just beginning [36].

### Community-Based Learning Collaborative (CBLC) implementation model

One limitation of the LC model is its limited focus on providers from multiple professional disciplines and agencies across service settings. For example, while the LC emphasizes training of mental health providers in an EBT, its focus is typically on teams from single mental health agencies. While this increases the supply of trained mental health clinicians, it has limited impact on the overall community service systems for children because the LC does not specifically include strategies to increase awareness and demand for a particular EBT among the broader array of community agencies and professionals that serve children, such as child welfare, schools, or juvenile justice.

**Fig. 1:**
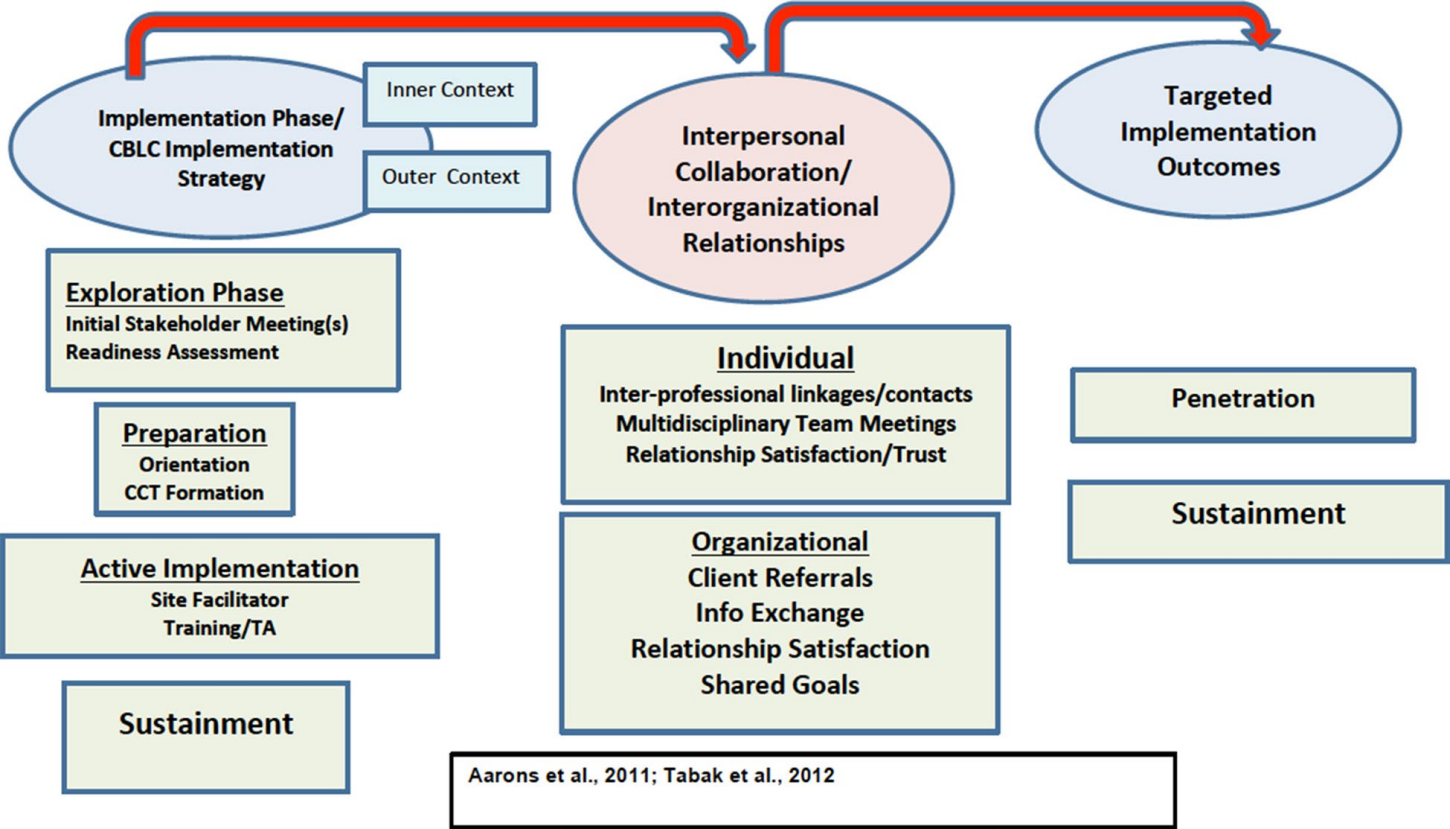
CBLC implementation

The CBLC model is an adaptation of the LC model that includes an expanded focus on community service systems to promote the adoption, ongoing use (i.e., penetration) and sustained use of EBTs. Specifically, CBLCs extend the LC model in three important ways. First, the CBLC model includes conjoint training of clinical and broker (i.e., nonclinical professionals who identify, refer, and monitor children and families in need of mental health services) professionals from multiple organizational levels (i.e., front-line providers, supervisors, and senior leaders) and from multiple service systems (i.e., child welfare, juvenile justice, and mental health) within a targeted community to build both the supply and demand for EBTs. Second, CBLC activities include a broker training curriculum for specific skills focused on screening, development of treatment and service plans, referrals for additional services when warranted, and ongoing case monitoring; this curriculum is delivered during breakout sessions as part of the aforementioned conjoint training. Finally, CBLC activities are designed to develop and sustain use of skills and interprofessional collaboration (IC) strategies following training sessions. For example, trainers conduct consultation calls with clinical providers (bimonthly), child welfare providers (monthly), and senior leaders (monthly) over the course of the 12-month CBLC, and participants must attend a specified number of calls that varies across roles to successfully complete the training (i.e., 12 for clinical providers; six for brokers and six for senior leaders). Figure 1 depicts the CBLC model and its hypothesized linkages to IC and interorganizational relationships (IOR) and targeted EBT implementation outcomes, including penetration, defined as the use of the targeted EBT among participants, and sustainment (i.e., on-going, long-term use following training). Table 1 delineates the specific implementation strategies that comprise the CBLC and the intended purpose of each strategy.

**Table 1:** CBLC implementation model

CBLC strategy/activity by implementation phase	Purpose
*Exploration/preparation*	
1.1* Senior leader stakeholder meetings* (conference calls and in-person): overview of CBLC; identification of key stakeholders; 1.2 *Community Change Team* (CCT) formation 1.3 *Readiness assessment* agency self-study; key stakeholder phone interviews 1.4 *Orientation*	1.1 Early planning and consensus building; create/highlight shared goals and resources; establish/strengthen cooperative interactive relationships; identify potential change agents/opinion leaders; information dissemination 1.2 Foster inter-organizational relationships; opportunities for shared brainstorming/problem-solving; increased communication 1.3 Identify service gaps, organizational capacity; availability of resources; existence/quality of coordinated care across agencies; potential implementation barriers 1.4 Provide overview of CBLC (information dissemination)
*Active implementation*	
2.1 *Pre*-*work* registration; pre-CBLC on-line assessment; readings; completion of on-line web courses (TF-CBTWeb; clinicians; CVWeb brokers) 2.2 *In*-*person training sessions*: track training (clinicians, brokers, senior leaders); community change team (CCT) activities (2 or 3, 2-day sessions)	2.1 Assess baseline knowledge; assess individual and organizational factors related to implementation outcomes 2.2 Build supply/demand concurrently; facilitate knowledge/skill acquisition in TF-CBT and case management/monitoring activities; define and reinforce professional roles and responsibilities; further development/strengthening of CCT
*Action periods*	
(3, 3–4 month periods): treatment implementation; weekly/monthly clinical and broker metrics; phone consultation	Training/technical assistance; identification of implementation barriers and strategies to address barriers; tracking of TF-CBT use/self-reported competence; tracking broker case management/case monitoring activities
*Sustainment*	
*Post CBLC assessment period*: monthly clinical/broker metrics; participant interviews	Assess continued use of TF-CBT; broker use of case management/monitoring activities; involvement in CCT

While there has been a proliferation of implementation research, relatively little has focused on trauma-focused EBTs targeting child welfare populations, and there remains a gap in our knowledge regarding the effectiveness of existing implementation models to guide these efforts. Studies indicate that coordination between child welfare and mental health service providers increases mental health service utilization [29, 37, 38], which may improve children’s mental health [35, 39, 40]. Limited research has examined specific implementation strategies to enhance and sustain IORs that would support delivery of trauma-focused EBTs for children in the child welfare system. Thus, the aims of this research study are to examine whether CBLC strategies are associated with (1) increased penetration of TF-CBT and broker case management strategies over the course of the CBLC (phase 1) and sustainment of practices throughout the phase 2, 18-month follow-up period; and (2) increased IC and IOR between child welfare and mental health service agencies over the course of the CBLC (Phase 1) and sustainability of these relationships, as measured throughout the Phase 2 follow-up period. Additionally, an exploratory aim is to examine clinician fidelity to TF-CBT and its association to clinical outcomes (phase 1).

## Methods/design

### Study design overview

This observational, mixed-methods study involves two phases. Phase 1 includes analyses of existing prospective quantitative and qualitative quality improvement and project evaluation data collected during eleven CBLCs conducted as part of Project BEST (Bringing Evidence-Supported Treatments to South Carolina children and families), a South Carolina statewide initiative, funded by the Duke Endowment, to implement TF-CBT. Data were collected from participants, before and immediately after each CBLC, and weekly and monthly throughout each CBLC. In addition, clinical participants were required to complete TF-CBT treatment with a minimum of two cases, which included administration of pre- and post-treatment assessment measures as a way to collect preliminary treatment outcome data. Phase 2 involves collection of new quantitative and qualitative data over an 18-month period to evaluate changes in relations among IC, IOR, social networks and the penetration and sustainment of TF-CBT in targeted communities. [(Note. The phase 2, 18-month data collection occurs over the course of the currently funded grant period. Since the CBLCs were conducted over a 5-year time period, the time since CBLC completion varies from 0 to 5 years (the most recent CBLC ended in January 2016)]. Both phases of the project received ethics approval by an institutional research review committee.

### Participant recruitment

Phase 1 involves secondary analysis of data collected from 998 individuals who participated in one of the selected Project BEST CBLCs. This included *n* = 570 (57.1 %) clinicians, *n* = 268 (26.9 %) brokers and *n* = 160 (16.0 %) senior leaders. Clinical participants identified *n* = 2361 training cases; pre-treatment assessments were obtained on 1664 (70.5 %) cases, and post-treatment assessments on 908 cases (54.5 % of those with pre-treatment assessments).

For phase 2 data collection activities, all Phase 1 participants (*N* = 998) are eligible for inclusion, whether or not they completed all training requirements. Those individuals who are not currently employed in a mental health or broker service organization will have the opportunity to complete the initial phase 2, Time 1 survey described below, but will not be eligible for the ongoing phase 2 activities. Targeted enrollment for Phase 2 is *n* = 150, with efforts to recruit equal numbers of clinicians, brokers, and senior leaders (*n* = 50 each). All participants who attended the initial training session of a completed CBLC will be notified of the research project through a combination of email, letter, and telephone contacts. Senior leaders from participating agencies will be asked to assist with recruitment of current staff and to reach out to individuals who have left the organization at which they were employed during the CBLC (i.e., snowball sampling).

In addition to general participation in phase 2, a subset of senior leaders, clinicians and brokers (*n* = 15 each), stratified using purposive sampling procedures [41–43], will be selected to complete key informant interviews. A list of potential informants will be generated from the population of individuals who participated in the Project BEST CBLCs. A combination of phone, email, and mail recruitment strategies will be used to contact these individuals. We will make up to three email and phone attempts to reach potential participants and will track our efforts (e.g., never reached, refused participation, etc.). If an individual is unavailable to complete an interview, we will move to the next person on the list until we have completed the targeted number of interviews. Participants will be paid for their time ($25/interview), and interviews will be conducted via phone or in-person depending on participant preference. As further incentive, all participants in phase 2 will be offered the opportunity to participate in a no-cost ‘booster’ training to provide additional instruction in clinical and/or broker-related skills taught in the initial CBLC (specific topics will be determined based on participant preference).

### Measures

#### CBLC components

The CBLC implementation checklist (see Table 2) will be used to assess participant and faculty adherence to components of the CBLC model. This assessment documents whether each key CBLC activity is completed, the date of completion, and when each participant completes all core CBLC activities for his/her role. Together, these events and activities form a set of indicators for the degree to which CBLC components were implemented with each participant. Furthermore, the use of this instrument will enable us to collect preliminary data on the relationships between completion of each of the CBLC activities at the group and individual levels and our measures of IC, IOR and use of TF-CBT. In addition, the post-CBLC evaluation includes questions that assess how useful each of the CBLC components were in building and supporting IC/IORs, as well as use of TF-CBT (see Tables 2, 3, 4).

**Table 2:** CBLC implementation checklist

Phase	Component	Strategy	Party responsible	Completed (y/n)	Date of completion	Tracked for each participant
Exploration/preparation	Stakeholder meetings	Phone	CBLC faculty			Yes/no
		In person	CBLC faculty			Yes/no
	CCT formation	Phone/in-person	Senior leaders			Yes/no
	Participant/team selection	Phone/in-person	CBLC faculty/senior leaders			
Preparation	Readiness assessment	Agency self-study	CAC ED and CCT			
		Key stakeholder interviews	CBLC faculty			
		Pre-CBLC on-line assessment	Participants			Completed—yes/no
	Orientation	In person	CBLC faculty			Attended (y/n)
	Pre-work activities	On-line registration/assessment	Participants			Completed (y/n)
Active implementation	LS1/2—in person training sessions	Track training (clinicians, brokers, senior leaders)	CBLC faculty			Attended (y/n)
		Community change team activities (e.g., PDSAs)	CBLC faculty			
	Action period	Clinical consultation calls	Expert faculty			# of Calls
		Broker consultation calls	Expert faculty			# of Calls
		Senior leader consultation calls	Expert faculty			# of Calls
		Clinical case identification/registration	Participants			# of Cases
		Client pre-treatment assessment	Participants			
		Clinical metrics—weekly/monthly	Participants			% completed
		Broker metrics—monthly	Participants			% completed
		Senior leader metrics-monthly	Participants			% completed
	Post CBLC	Evaluation	CBLC faculty			Yes/no

**Table 3:** Study constructs and measurement

Construct	Level of analysis	Data collection methods	Variables/items measured	Time of assessment
	Individual	On-line survey^a, b^	Demographics; knowledge/attitudes towards EBTs; clinical/broker practices; organizational characteristics	*Phase 1* pre/post CBLC *Phase 2* 2 time points: Y1Q3; Y2Q3
Interpersonal collaboration (IC) (phase 1 and phase 2)	Individual	On-line survey^a, b^	Clinician IC (# of contacts with another CBLC professional regarding assessment/treatment services, # of times attended a multidisciplinary team meeting) Broker IC (# of times broker contacted professional for assessment information/treatment progress, # of cases taken to MDT, # of children referred to a CBLC therapist, # of children in caseload receiving TF-CBT) Provider attitudes towards collaboration Barriers towards collaboration	*Phase 1* monthly during active implementation *Phase 2* monthly
Inter-organizational relationships (IOR) (phase 1 and phase 2)	Social network analysis	Participant online social network survey^a, b^	*Provider networks* Identify up to five individuals on whom you rely for advice about whether and how to use EBTs for meeting the mental health needs of youth served by your agency; Identify up tofive individuals to whom you seek professional advice on youth with a trauma history specifically	*Phase 1* mid CBLC *Phase 2* 2 time points: Y1Q3; Y2Q3
		Senior leader online survey^a, b^ Senior leader interview^a, b^	*Organizational networks—link content* Client referrals Coordination Information exchange Relationship satisfaction Shared goals Formalized agreements *Organizational networks—qualitative data* Facilitators/barriers towards collaboration	*Phase 1* mid CBLC *Phase 2* 2 time points: Y1Q3; Y2Q3 *Phase 1* mid CBLC *Phase 2* 2 time points
Penetration (phase 1 and phase 2)	Individual	Weekly/monthly surveys^a, b^ Participant interviews^b^	# of clinicians using TF-CBT # of children receiving TF-CBT # of brokers using of case management/monitoring strategies # children monitored by brokers Facilitators/barriers towards provider use of TF-CBT and case management/monitoring strategies	*Phase 1* during active implementation^a^ Weekly for clinicians Monthly for brokers *Phase 2* 2 time points: Y1Q3; Y2Q3
	Organization	Senior leader interview^a, b^	# of organizations providing TF-CBT # of agency referrals for TF-CBT # of staff training in TF-CBT # of agencies providing TF-CBT supervision Implementation Facilitators/barriers	*Phase 1* mid CBLC *Phase 2* mid/post CBLC^b^
Sustainment: *phase 2* post CBLC follow-up period (Y1Q2–Y2Q4)	Individual	Monthly on-line survey^b^ Participant interviews	Clinician use/competence in TF-CBT Broker use of case management/monitoring strategies Facilitators/barriers towards provider use of TF-CBT and case management/ monitoring strategies; and collaboration	Monthly during sustainment^b^ 2 time points: Y1Q3; Y2Q3^b^
	Organization	Senior leader interview^b^	TF-CBT provision/ referral patterns Staff training TF-CBT supervision *IOR—social networks*: (see above)	2 time points: Y1Q3; Y2Q3^b^ Mid sustainment phase (Y2Q4)^b^

^a^Phase 1: Existing data

^b^Phase 2: New data

**Table 4:** Measures by project phase

Phase 1 measures	Phase 2 measures
A. Registration	M. Senior leader participant interview
B. Pre-work survey	N. Broker participant interview
C. Weekly/monthly clinical metrics	O. Clinician participant interview
D. Senior leader participant interview	P. Registration and time 1 survey
E. Provider social network survey	Q. Provider social network survey
F. Senior leader survey-organizational social network survey	R. Organizational social network survey
G. Supervisor weekly metrics	S. Clinical monthly metrics
H. Broker monthly metrics	T. Broker monthly metrics
I. Senior leader monthly metrics	U. Senior leader monthly metrics
J. Project BEST post evaluation	
K. Child/caregiver pre treatment packet	
L. Child/caregiver post treatment packet	

#### TF-CBT outcomes

As part of phase 1, clinical providers (*n* = 570; 57.1 % of the CBLC participants) were asked to identify a minimum of five TF-CBT training cases from their usual caseloads, with the goal of completing the full TF-CBT protocol with at least two cases. For each training case, clinicians were required to conduct pre-treatment and post-treatment assessments, using standardized measures of post-traumatic stress disorder (PTSD) and depression, to collect preliminary data on treatment outcome and its relationship to provider fidelity to TF-CBT. The *University of California*—*Los Angeles (UCLA) PTSD Reaction Index for DSM*-*IV Parent, Child, and Adolescent* [44] served as a brief self- or parent/caregiver-report screening tool to obtain information regarding trauma exposure and PTSD symptoms. The *Short Moods and Feelings Questionnaire* [45] is a brief self-report measure of depression completed by the child and (separately) a caregiver. As of February 2013, we discontinued use of the UCLA PTSD Reaction Index in Project BEST CBLCs due to a newly imposed cost for use of the measure. Since sustaining the use of standardized measures in everyday practice after the completion of the CBLC is a major goal of our implementation efforts, we replaced the UCLA PTSD Reaction Index in subsequent CBLCs with a brief trauma history screen, developed by the Harborview Sexual Assault Treatment Center in Seattle Washington, and the *Child PTSD Symptom Scale* [46].

#### TF-CBT fidelity

While observational coding systems are generally regarded as the ‘gold standard’ for determining treatment fidelity [47–49], recent research suggests that observational methods may not be superior to therapist report, but instead that these two measurement methods yield different types of data (e.g., micro vs. macro) that have utility for different purposes [47–50]. As a feasible and pragmatic approach for community practice settings, we elected to assess fidelity via clinician self-report. During Phase 1, clinical providers completed a weekly on-line checklist about each of their TF-CBT training cases in which they rated: (1) whether or not the child and the caregiver participated in treatment that week (*dosage*); (2) the specific components of TF-CBT that were used that week (*adherence); and* (3) their perceived competency in delivery of the TF-CBT component(s) delivered that week (*competence).* These weekly clinical metrics were modeled after the *TF*-*CBT Practice Checklist*-*Self Report* [51], which has demonstrated adequate levels of internal consistency reliability in prior research [23]. Duration of treatment was derived from the completion dates of the pre and post-treatment assessments that were administered to all training cases. Additionally, in phases 1 and 2, all participating clinicians reported on their use of TF-CBT, whether their agencies provide TF-CBT, and whether they received supervision in TF-CBT (*Pre/Post CBLC evaluation*). Finally, on a weekly basis throughout phase 1, participating clinical supervisors reported on the number of clinicians to whom they provided supervision on TF-CBT cases, number of TF-CBT cases for which they provided clinical supervision, and time spent in supervision on each of the TF-CBT components.

#### Interprofessional collaboration (IC) (see Table 3)

Based on extant literature [28, 29, 31, 40, 52–59], key indicators of IC include measures of communication and information exchange between professionals within and across agencies. We will measure IC using existing quantitative quality improvement and program evaluation measures reported by participants on a monthly basis via online survey throughout Phase 1. Measures reported by clinicians and brokers include (1) number of contacts with another CBLC professional regarding assessment or treatment information and (2) number of times the clinician attended a multidisciplinary team (MDT) meeting. Indices of IC reported only by broker participants include (1) number of children referred to a CBLC therapist, (2) number of children on their caseload receiving TF-CBT, and (3) number of times they discussed a client’s treatment progress with the treating therapist.

#### Inter-organizational relationships (IOR) (see Table 4)

Separate measures will assess social networks at two levels: individual providers and organizations. Provider social networks yield information about the linkages between professionals within and across agencies and can help to identify individuals who may be key opinion leaders or change agents within a given community (i.e., regardless of their specific ‘home’ agency, and including private practitioners who are not affiliated with a particular agency). Organizational social networks help to identify agencies that are most pivotal in facilitating coordinated service provision across providers, which is useful given anticipated employment mobility (i.e., turnover) among individual providers.

##### Provider social networks

During phase 1, participants were administered a two item survey during the CBLC learning sessions to assess existing provider social networks. Participants were asked to (1) name as many as five individuals to whom they have turned to for professional advice about youth with trauma histories and how frequently they communicated in person, on the telephone, or via email; and (2) name up to five individuals that they contact regarding the care and coordination of services for children and families who have experienced abuse, with ‘contact’ defined as instances in which any of the following occurs (via in person, phone, or email): sharing or exchange of information, consultation, or coordination of efforts across agencies related to assessment, treatment and/or referral. This survey was intentionally left unbounded (i.e., respondents were not restricted to naming only those participating in the current CBLCs) to examine whether the CBLCs were ‘missing’ key individual stakeholders that could inform our planning efforts for future implementation efforts.

##### Organizational social networks

During phase 1, an on-line survey was administered to participating senior leaders (*n* = 24), or an agency representative (*n* = 40) for those agencies that did not have a senior leader participant, at the end of each CBLC. A total of *n* = 62 (97 %) of the surveys were completed. The survey design was based on prior research [40, 52, 54–57, 60] on identifying and assessing organizational social networks and IORs in physical and mental health care. Respondents were provided a bounded list of participating CBLC agencies and asked to name up to 10 with whom they have consistent contact (at least 1/month) regarding the care and coordination of services for children and families who have experienced abuse. The definition of “contact” was the same as described above.

#### Penetration and sustainment (see Table 3)

Existing weekly clinician online surveys administered during phase 1 assessed *penetration* by asking about clinician use of TF-CBT, and their perceived competence in delivering TF-CBT components. Relatedly, existing monthly broker online surveys asked about broker use of treatment planning and case management/monitoring skills. In phase 2 (see Table 4), we will continue to collect data on clinician use/self-reported competence in TF-CBT and broker use of case management/monitoring skills over the post-CBLC follow-up period.

#### Phase 2 participant interviews

During phase 2, qualitative data will be collected from key informant interviews (N = 45). Interview schedules were developed for each participant role (i.e., Clinician, Broker, and Senior Leader) to measure key constructs related to IC/IOR and implementation identified in the extant literature. Additional questions will assess the frequency, nature, and quality of contacts among professionals both during and following completion of the CBLC, as well as facilitators and barriers to collaboration. These interview data will be used to obtain additional details and context for the aforementioned quantitative data on IC and IOR. Similarly, quantitative data regarding penetration and sustainment will be supplemented with qualitative interview data about the CBLC strategies, as well as facilitators and barriers to sustaining learned practices over time.

Once the interviews are completed, each interview will be assigned to one of two bachelor’s level coders. Coders will be trained in a group format through didactic instruction and discussion of the interviews and coding manual. The procedures for coder training and quality assurance are informed by those used by the research team in prior studies. Reliability coefficients and other coder statistics will be calculated on a routine basis, and this information will be used to guide supervision and (if necessary) re-training. Approximately 20 % of interviews will be double-coded for reliability purposes. Weekly coder consensus meetings will be held during the interview period to maintain a high level of fidelity to the coding system.

### Data analysis

Once the interview transcripts have been coded, the computer program QSR NVivo [61] will be used to generate a series of categories arranged in a treelike structure connecting text segments grouped into separate categories of codes or “nodes.” These nodes and trees will be used to further the process of axial or pattern coding [62] to examine the association between different a priori and emergent categories. They also will be used in selective coding of material to identify the existence of new, previously unrecognized categories. The number of times these categories occur together, either as duplicate codes assigned to the same text or as codes assigned to adjacent texts in the same conversation, will be recorded, and specific examples of co-occurrence illustrated with transcript texts. Through the process of constantly comparing these categories with each other, the different categories will be further condensed into broad themes [63].

Mixed quantitative/qualitative data will be collected and analyzed sequentially for three distinct purposes [52] (see Table 5). First, *expansion* analyses will use data from *n* = 45 key informant interviews to provide further explanation of the quantitative findings related to CBLC strategies and activities (i.e., explanations of observed trends in the quantitative results). For example, the interviews will be used to expand data from the phase 2 monthly quantitative surveys to explain possible reasons for relationships between CBLC strategies and penetration of TF-CBT. Second, *convergence* analyses will examine the extent to which interview data support the quantitative monthly online survey data (i.e., validity of the quantitative data). Finally, *complementarity* analyses will enable us to examine both quantitative and qualitative data to explore further factors related to sustainment of IC/IOR and penetration/use outcomes over the follow-up period. Taken together, the results of these analyses will inform further refinement of the CBLC model.

**Table 5:** Relationship between quantitative and qualitative data

Structure	Function	Research question(s)	Quantitative	Qualitative
Quan→QUAL	Expansion	What specific CBLC components are most helpful/successful and what are the barriers to activity completion?	Participation in CBLC activities (e.g., training sessions, consultation calls; CCT participation)	Post CBLC stakeholder interviews (examine CBLC *process*): *What specific activities were most/least helpful to you as a provider? To your agency?*
QUAN→qual	Convergence	What CBLC strategies are associated with increased IC and IOR intensity?	Monthly on-line surveys assessing # of contacts with other professionals related to assessment, referral and treatment services	*What activities/parts of the CBLC helped to facilitate relationships and collaborations between agencies in the CBLC?*
QUAN + QUAL	Complementarity	What CBLC strategies are associated with sustained IC/IOR?	Monthly on-line surveys assessing # of contacts with other professionals related to assessment, referral and tx services during sustainment phase	*What activities/parts of the CBLC helped to sustain relationships and collaborations amongst professionals/between agencies participating in the CBLC? What was the role of the CCT?*

#### Aim 1: relationships between CBLC strategies and penetration/sustainment

Table 1 provides an overview of CBLC strategies, their purposes, and the corresponding phases of implementation. Participation in each activity is documented with the CBLC Implementation Checklist (Table 2), which will permit examination of relations between these strategies and penetration/sustainment indices. *Penetration* will reflect data collected during phase 1, and *sustainment* will reflect outcomes occurring during phase 2 (see Table 3). Descriptive statistics will be used to examine the key CBLC strategies and penetration/sustainment indices for clinicians and brokers, and to evaluate the magnitude and direction of associations among these indicators; single- and multi-level regression-based analyses will be used. Most of the indicators are measured longitudinally, leading to a two-level data structure with repeated measurements of penetration or sustainment indicators (level-1) nested within participants (level-2). Accordingly, these data will be analyzed using mixed-effects regression models (e.g., Raudenbush and Bryk [64]) implemented in HLM software [65]. As one example, *for clinicians*, the number of consultation calls (a CBLC strategy) will be entered as a level-2 predictor of the repeated measurements of the number of children receiving TF-CBT per month (aggregated from clinician self-reports) (index of penetration). A similar approach will be used for *Broker* outcomes. For example, the number of children screened for trauma or PTSD across each of the 12 months will be specified as the longitudinal *penetration* outcome, and at level-2, the predictor would be the number of consultation calls attended. Associations between CBLC strategies and *sustainment* will be investigated utilizing a series of regression models that are consistent with those just described for penetration, but will cover the Phase 2 follow-up period rather than the Phase 1 active implementation period. We will also evaluate the predictive validity of our TF-CBT self-report fidelity measure with respect to positive treatment outcomes on the PTSD and depression measures for TF-CBT training cases.

#### Aim 2: relationships between CBLC strategies, IC, and IOR

Existing quantitative and newly collected quantitative/qualitative data will be used to test whether CBLC implementation strategies (Table 1) are associated with increased IOR and IC intensity during phase 1 and phase 2. For example, analyses will be conducted to examine the association between participation in training sessions (i.e., CBLC Active Implementation strategy) and the number of contacts with professionals regarding referral, assessment, or treatment of children (IC).

The IOR measures described above will yield data on six different types of inter-organizational networks (client referrals, coordination, information exchange, relationship satisfaction, shared goals, and formalized agreements). The matrix of ties used to analyze advice networks will be constructed from data collected from the web-based survey, supplemented by data collected during the qualitative interviews [66]. The social network analysis will proceed in three stages: network visualization (using NetDraw 2.090), structural analysis (using UCINET for Windows, Version 6 [67]), and statistical analysis of outcomes. Several network level measures of structure will be assessed, including: total number of ties, network size, density (i.e., the number of reported links divided by the maximum number of possible links), average distance between nodes, and the number of components (i.e., unique sub-networks) [68]. To assess status and interconnectivity within the network, we will calculate degree centrality for incoming and outgoing ties. We will also examine several other measures of network status, including between-ness, closeness, and eigenvector centrality. To examine homophily (i.e., likeness between individuals in a network based on specified criteria), data will be assessed based on service sector (e.g., mental health, child welfare, juvenile justice) and CBLC. For each service sector and each network, descriptive statistics (e.g., means, standard deviations) will be used to examine the average strength of IORs. Within each service sector, we will also conduct Pearson correlational analyses to assess the degree of overlap between the six types of networks. To test the significance of the correlations, we will employ quadratic assignment procedure to account for non-independence in the network data [66, 69].

To examine whether CBLC implementation strategies are associated with increased *IOR*, we will conduct paired samples t-tests comparing pre and post-test measures (from Phase 1) of network density for each of the six types of IORs (i.e., client referrals, information exchange, coordination, relationship satisfaction, shared goals, and formalized agreements). Due to the non-independence of the network data, these analyses will be conducted in UCINET 6 [67] using a bootstrap approach to estimate standard errors [70]. For each type of IOR, network density will be calculated on the valued data, and ranges from 0 (no organizations have a relationship) to 1 (all organizations have the strongest possible relationship).

### Attrition and missing data

Because some data will inevitably be missing due to attrition, the methods recommended by Schafer and Graham [71] will be used to evaluate missing data assumptions and guide the subsequent analyses. Given few missing data and evidence supporting a missing at random mechanism, maximum likelihood-based estimation procedures will be utilized with the available data. Given non-trivial missing data and evidence supporting a missing at random mechanism, multiple imputation for longitudinal data will be used to provide complete data [72]. Finally, given non-trivial missing data and evidence suggesting that data are not missing at random, pattern mixture models will be used to control the missing data patterns [73].

### Study status

We are currently in the process of analyzing Phase 1 study data, have completed recruitment for phase 2 activities (*n* = 162), and have been collecting monthly metrics to examine IOP/IC and sustainment of trauma-focused practices. We have also completed the *n* = 45 qualitative interviews (*n* = 15 senior leaders, *n* = 16 clinicians, *n* = 14 brokers) and these are now being transcribed for coding purposes. Development of the coding manual and coder training are in the initial stages.

## Discussion

An ongoing challenge facing the mental health and child welfare systems is to determine the most efficient ways to implement EBTs relevant to children involved in the child welfare system in community service agencies so they are readily available, accessible, and sustained. Rigorous research has identified a number of EBTs to address mental health problems, such as those related to violence exposure. However, universal access to these services is still not available, especially among traditionally underserved minority populations that are disproportionately represented in the child welfare setting. While research indicates that coordination between child welfare and mental health service providers increases mental health service utilization, which may improve children’s mental health, to our knowledge, the present study represents the first effort to examine the effectiveness of specific implementation strategies to build and strengthen relationships between the multiple mental health and child welfare professionals involved in service provision for children. The results of the present study will inform development of a quasi-experimental clinical trial that will use a time series design to evaluate the effectiveness of the CBLC (vs. training as usual) as a mechanism to build IC/IORs and thereby achieve greater penetration and sustainment of EBTs for children.

## Limitations

A distinct advantage of this study is that, for the purposes of creating generalizable knowledge, it leverages an ongoing state and foundation funded initiative designed to increase access to effective services for children. Study limitations associated with capitalizing on that initiative include the lack of an experimental or quasi-experimental design (because a comparison condition is not available), which precludes causal statements regarding the relations among elements of the CBLC and implementation outcomes. There is also likely to be wide variability in participant and agency characteristics, such as variable representation from service systems, unequal numbers of clinical, broker and senior leader participants within each CBLC, and other idiosyncratic factors within individual communities. Our analyses will take into account these nested data, and importantly, a core component of this study is the ability to test feasibility of the CBLC and explore these variable factors to inform development of a large-scale effectiveness study. The level of statistical power is somewhat limited given the stage of the research; importantly, however, the proposed sample sizes are sufficient for obtaining accurate estimates of the effects of interest. A final limitation is the use of a self-report method to measure therapist adherence and competence. We elected to use this measurement system because of concerns related to increasing the potential burden for participating clinicians and our intent to introduce measurement strategies that could potentially be sustained over time.

## Conclusions

Since LCs are being widely used as a way of implementing EBTs across agencies and targeted communities, it is important for research to examine the effectiveness of these implementation strategies. As noted, while EBTs exist for youth, access and availability are not universal, meaning that many are not receiving needed services. Of particular relevance for this study, violence exposure remains high among youth, particularly among those involved in child welfare. The CBLC offers the distinct opportunity to integrate training for the multiple service providers and service agencies involved in the care of trauma-exposed youth and their families. This study aims to evaluate the role of the CBLC in strengthening IC and IOR, mechanisms hypothesized to be important to increasing the penetration and sustainability of EBTs. Findings also may assist in the creation of knowledge and resources that will benefit other communities who wish to engage in similar training and implementation efforts.

## Abbreviations

EBT: evidence-based treatment
TF-CBT: Trauma-Focused Cognitive Behavioral Therapy
LC: Learning Collaborative
CBLC: Community-Based Learning Collaborative
IC: interprofessional collaboration
IOR: interorganizational relationships
Project BEST: bringing evidence-supported treatments to South Carolina children and their families
PTSD: post-traumatic stress disorder
UCLA: University of California—Los Angeles
